# Optimization and comparison of different methods for RNA isolation for cDNA library construction from the reindeer lichen *Cladonia rangiferina*

**DOI:** 10.1186/1756-0500-2-204

**Published:** 2009-10-05

**Authors:** Sini Junttila, Kean-Jin Lim, Stephen Rudd

**Affiliations:** 1 Centre for Biotechnology, Tykistökatu 6 A, 20520 Turku, Finland

## Abstract

**Background:**

The reindeer lichen is the product of a mutualistic relationship between a fungus and an algae. Lichen demonstrate a remarkable capacity to tolerate dehydration. This tolerance is driven by a variety of biochemical processes and the accumulation of specific secondary metabolites that may be of relevance to the pharmaceutical, biotechnology and agriculture industries. These protective metabolites hinder *in vitro *enzymatic reactions required in cDNA synthesis. Along with the low concentrations of RNA present within lichen tissues, the process of creating a cDNA library is technically challenging.

**Findings:**

An evaluation of existing commercial and published protocols for RNA extraction from plant or fungal tissues has been performed and experimental conditions have been optimised to balance the need for the highest quality total ribonucleotides and the constraints of budget, time and human resources.

**Conclusion:**

We present a protocol that balances inexpensive RNA extraction methods with commercial RNA clean-up kits to yield sufficient RNA for cDNA library construction. Evaluation of the protocol and the construction of, and sampling from, a cDNA library is used to demonstrate the suitability of the RNA extraction method for expressed sequence tag production.

## Background

Lichen are formed as a product of the symbiotic relationship between a fungus and its photosynthetic partner, which can be either an algae or a cyanobacterium [1]. The fungus, or mycobiont, forms a three-dimensional vegetative structure called a thallus, within which the photosynthetic partners, or photobionts, are located. The algal cells comprise only approximately 7% of the total thallus volume [2].

Approximately one-fifth of fungal species form obligate symbiotic associations with green algae or cyanobacteria. This increases to about 46% for the ascomycete fungi and thus the processes of lichenisation are critical to the understanding of both ascomycete relationships and the evolution of mechanisms for the control and maintenance of plant-fungal interactions. An estimated 100 species of algae from 40 genera are reported to form lichen symbioses [3].

The molecular nature of the symbiosis is still under discussion; some researchers observe a controlled parasitism of the photobiont by the mycobiont [1], while others see a more mutualistic relationship [4].

Regardless of the nature of the relationship, lichens inhabit some of the harshest climates on earth, and have demonstrated a capacity to at least survive the more challenging extremes of space [5]. Lichen are poikilohydric and lack the ability to actively control the water content within their thalli; they have as a consequence evolved physiological mechanisms to tolerate frequent cycles of desiccation and wetting. Their remarkable ability to survive even anhydrobiosis allow lichen species to flourish, albeit slowly, in some of the driest ecological niches. The anhydrobiotic state is driven by the accumulation of specific metabolites and polysaccharides that limit the damage caused by desiccation, and maintain sufficient physiological integrity that such damage can be repaired upon rewetting [6,7]. The characterisation of these molecular mechanisms has potential agronomic value, but there are few resources available that may facilitate the characterisation of these processes. Genetic resources have been established for the systematic study and classification of lichen, (reviewed in [3]). Lichens have not yet been investigated within a genomics context, and there is little in the way of genome sequence data available for any lichen species.

The availability of methods for the purification of high quality ribonucleic acids (RNA) is essential for studies aimed at constructing cDNA libraries or performing array-based hybridisations where microgram quantities of concentrated and chemically pure mRNA may be required. RNA isolation in lichen is complicated by the abundance of protective secondary metabolites and polysaccharides, chemicals known to interfere with RNA isolation protocols that lower both the yield and quality of the extracted total RNA [8]. In addition slow growing lichen tissues contain modest concentrations of mRNA, and as a result a larger mass of starting tissue, and multiple extraction steps are required to purify sufficient RNA.

It is the aim of our research to develop genomic resources for the fructicose lichen, *Cladonia rangiferina*. We have evaluated and optimised a process for RNA isolation from the secondary metabolite rich tissues of *Cladonia rangiferina *that balance the needs of sample purity for downstream library construction, cost and labour requirements.

## Methods

### Sample collection and pre-treatment

Clumps of *Cladonia rangiferina *were collected from the island of Kuusisto in Kaarina, Finland. The lichen was cleaned and stored desiccated at -20°C as this has been found to be the best way to store lichen samples [9]. Prior to RNA isolation the dry lichen material was weighed and rewetted with tap water overnight. The samples were powdered in liquid nitrogen using mortar and pestle.

### Sigma Spectrum Plant Total RNA kit

RNA extraction was performed according to the manufacturer's instructions (Sigma Aldrich, USA). The amount of starting tissue used was 100 mg of lichen tissue.

### TRIzol reagent

An extraction procedure was modified from the manufacturer's (Invitrogen, USA)

instructions by adding an additional chloroform extraction step after the first chloroform extraction and replacing the isopropanol precipitation with ethanol precipitation at -80°C for 2 hours. Different lichen tissue/TRIzol reagent ratios were evaluated with 1 g of lichen tissue in a 15 ml volume of TRIzol reagent being optimal.

### Dong & Dunstan

The extraction protocol was modified from [10] by replacing the LiCl precipitation with ethanol precipitation at -80°C for 2 hours. 1 g of lichen tissue was added to 15 ml of extraction buffer (50 mM Tris-HCl pH 8.0, 300 mM NaCl, 5 mM EDTA, 2% SDS, 0.5% polyvinylpyrrolidone MW 360 000) added with 0.5 mM aurintricarboxylic acid and 14.3 mM β-mercaptoethanol. After a 10-minute incubation at 65°C and centrifugation, 0.7 ml of 3 M potassium acetate (pH 4.8) was added to the supernatant followed by incubation on ice for 30 minutes and centrifugation. The supernatant was precipitated with ethanol instead of LiCl, dissolved in water and extracted once with phenol and twice with phenol-chloroform-isoamylalcohol (24:23:1). The RNA was precipitated with ethanol and dissolved in water.

### CTAB + RNeasy Midi kit

The CTAB protocol was modified from Gooding, et al [11], who had modified the pine tree RNA extraction method [12]. 1 g of lichen tissue was added to 10 ml of pre-warmed (65°C) extraction buffer (2% CTAB, 2% polyvinylpyrrolidone MW 40 000, 200 mM Tri-HCl pH 8.0, 25 mM EDTA, 2 M NaCl, 0.5 g/l spermidine) added with 2% β-mercaptoethanol. The mixture was extracted twice with phenol-chloroform (1:1) and the supernatant was added to an equal volume of NTES buffer (1 M NaCl, 0.5% SDS, 10 mM, Tris-HCl pH 8.0, 1 mM EDTA) and chloroform (1:1). The RNA was precipitated with ethanol and dissolved in water. The extracted total RNA was cleaned using the RNeasy Midi Kit (Qiagen, Germany) according to the manufacturer's instructions. Approximately 20 samples were pooled into one clean-up column.

### Quality assessments

The extracted total RNA samples were stored at -80°C and concentrations were measured spectrophotometrically in Tris-EDTA buffer solution using the NanoDrop instrument (NanoDrop Technologies, Wilmington, USA). The purity of the total RNA was assessed using the A_260/280 _and A_260/230 _ratios given by NanoDrop. Quality was also inspected visually following gel electrophoresis of denatured RNAs.

### cDNA library construction

mRNA was isolated from the total RNA with Nucleo-Trap mRNA kit (Macherey-Nagel, Duren, Germany). A phage cDNA library was constructed from the mRNA using the ZAP-cDNA^® ^Gigapack^® ^III Gold Cloning (#200450) cDNA library synthesis kit (Stratagene, La Jolla, USA) according to manufacturer's instructions. Size fractionation was achieved using gel electrophoresis. Gel slices corresponding of between 500-1000 bp and 1000-3000 bp in size were excised and gel purified cDNA was cloned into a phage library. cDNA library clones were sequenced on an ABI PRISM 3130xl Genetic Analyzer capillary DNA sequencer following a BigDye v3.1 (Applied Biosystems, Foster City, USA) labelling reaction.

### Sequence analysis

Computational analysis of the raw sequence data was performed using the openSputnik sequence analysis platform [13]. BLASTX [14] was used to compare the *Cladonia *sequences to the NCBI non-redundant protein sequence database [15]. BLASTX matches were filtered using an arbitrary cutoff of 1e-10 and sequences were assigned as plant or fungal homologs using the NCBI Taxonomy [16] mappings and custom scripts implemented in the R statistical language.

## Results

### Evaluation of competing methods for RNA isolation efficiency

We evaluated a panel of four robust methods (Sigma Spectrum kit, TRIzol reagent, Dong and Dunstan and CTAB) that yielded varying quantities of total RNA. The CTAB method was supplemented with a further RNA purification with a commercial RNA cleaning kit to yield a fifth method. The yield and purity of the extracted total RNA from these five RNA isolation procedures is presented in table 1.

**Table 1:** A comparison of different RNA isolation procedures

**Method**	**Average Yield (μg/g of dry tissue)**	**Standard Deviation of Yield (μg/g)**	**A_260/280_**	**A_260/230_**	**Estimated extraction time for 1 mg of total RNA (hours)**	**Estimated cost for extracting 1 mg of total RNA (Euro)**
Spectrum plant total RNA kit	31.2	22.4	2.01-2.05	2.00-2.27	54	979
TRIzol reagent	162.1	52.7	1.47-1.65	0.42-0.91	5	235
Dong & Dunstan	126.3	33.1	1.57-1.68	0.27-0.41	9	19
CTAB	69.3	19.4	1.40-1.56	0.58-1.01	9	27
CTAB + RNeasy	8.6	5.8	2.22-2.24	2.50-2.93	62	286

Shown is the method along with average nucleotide yield from starting material (μg/g) and the standard deviation from at least six extractions. The A_260/280 _and A_260/230 _ratios provide insight as to the quality of the resultant nucleotides - an extraction of sufficient quality for cDNA library construction should have ratio quality values of approximately 2.0. Also shown are estimates of time (h) and costs (Euro) required for the extraction of 1 mg of total RNA - this is a typically required amount for mRNA purification for cDNA library synthesis or for genome resequencing techniques.

The Sigma Spectrum plant total RNA kit produces high-quality total RNA with a good yield and a hands-on time of 30-45 minutes. The biggest drawback of the method is the limited amount of starting tissue (100 mg) that can be used per column. To obtain the needed 1000 μg of total RNA for the subsequent mRNA isolation for cDNA library construction, over 30 g of lichen tissue is required. This represents at least 300 extractions, which renders the method both time-consuming and prohibitively expensive.

With TRIzol reagent and Dong & Dunstan method the yield is superior to the other methods, but the purity of the extracted total RNA is insufficient. The A_260/230 _ratios are low and indicate the presence of polysaccharides and/or secondary metabolites. Modifying the TRIzol procedure by adding a second chloroform extraction step and replacing the isopropanol precipitation with ethanol precipitation slightly improved the A_260/280 _ratio while the yield remained similar. LiCl precipitation step was omitted from CTAB method and replaced with ethanol precipitation in Dong & Dunstan method as this step radically reduced the RNA yield. When the RNA extracted using the TRIzol reagent or Dong & Dunstan method was further purified with RNeasy clean-up kit, superior A_260/280 _and A_260/230 _ratios were obtained. When resolved by gel electrophoresis, the RNA extracted with TRIzol reagent appeared to be of better quality than that extracted according to Dong & Dunstan method (results not shown). TRIzol reagent is expensive, thus the projected volumes required would support the application of a more cost effective RNA extraction protocol as the Dong & Dunstan method appears to yield RNA of insufficient quality.

**Figure 1:**
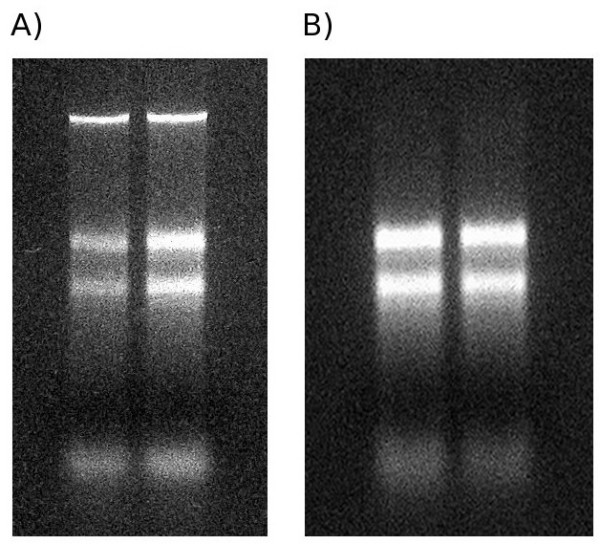
**Image showing nucleotide size distribution from CTAB (panel A) and CTAB + RNeasy RNA isolations (panel B)**. Nucleotides have been resolved by denaturing gel electrophoresis and have been stained using ethidium bromide and viewed under a UV transilluminator. In both samples, clear rRNA bands can be seen, suggesting the integrity of the RNAs. In the CTAB gel, a band of high-molecular weight DNA can be seen, this is absent from the CTAB + RNeasy extractions.

The CTAB method alone yields approximately half the amount of RNA compared to TRIzol reagent or Dong & Dunstan method, but with supplementary RNeasy clean-up the A_260/280 _and A_260/230 _ratios were suitable. Expensive chemicals are not required for the CTAB method, it can easily be scaled up if needed, with a hands-on time of around 4 hours for six samples. Total RNA extracted using the CTAB method contained considerable genomic DNA contamination. After purification with the RNeasy clean-up kit, which includes a DNase I treatment, the DNA contamination was no longer visible following denaturing gel electrophoresis (figure 1), but there was a marked decrease in RNA yield following the step. Although this combined method is labour intensive and uses much time, cost is a driving factor. The complete protocol is summarised in the Appendix.

A total of 1024 μg of total RNA from six CTAB+RNeasy RNA isolations were pooled, and the pooled ribonucleotides were used for construction of a phage cDNA library. Library titres of 2.4 × 10^6 ^and 3.1 × 10^6 ^PFU/ml for the 500-1000 bp and 1000-3000 bp fractions respectively were measured. The cDNA inserts of one 96 well plate from each size fractionated library were sequenced on a capillary DNA sequencer. The size of the lichen cDNA library sequences ranged between 200 and 800 bp (data not shown), with the majority of sequences falling in the range of 200 to 500 bp. BLASTX [14] analysis of the clustered consensus sequence following vector masking and CAP3 [17] EST clustering revealed that 70/170 (41%) of sequences match known sequences within the NCBI non-redundant protein sequence database (Additional file 1). Of these, 19 appear to match sequences of only fungal origin using the arbitrary expectation value of 1e-10. Four sequences appear to match plant sequences, but not fungal sequences. The sequence collection has been deposited in the dbEST database of expressed sequence tags under the GenBank accessions GH717691 - GH717859.

## Discussion

Little research has been performed on lichen at the molecular biological level and the available molecular resources for lichen species are focused towards taxonomic systematics and on the evolution of lichens. We have compared and optimised five different methods for extracting total RNA from *Cladonia rangiferina*.

The secondary metabolites present in lichen tissues interfere with conventional RNA extraction procedures and in the subsequent first strand cDNA synthesis. Using the mRNA from a standard RNA isolation/mRNA purification in cDNA library construction has resulted in disappointing titre counts (results not shown). It is essential, and ultimately cost-effective, to combine a conventional RNA isolation method with a commercial RNA clean-up kit to produce sufficiently clean RNA. Conventional methods allow for the amount of starting tissue to be scaled up as needed, such that impure total RNA can be extracted in large quantities. The conventional procedure best suited for lichen RNA isolation appears to be the CTAB method. The remaining polysaccharides and secondary metabolites are effectively removed in the following clean-up step, resulting in high quality total RNA with A_260/280 _and A_260/230 _ratios >2.

The overall yield from the optimised CTAB method with subsequent sample clean-up is less than 10 μg of total RNA per gram of starting tissue. This is much lower than the amount of RNA extracted from other model organisms, or the measured yields from the other methods (table 1). The RNA yield is lower than the commercial extraction kit, but is also less expensive. Extracting high quality total RNA from slow growing organisms with their low RNA concentrations is complicated. The need for aggressive cleaning of a first "dirty" RNA extraction leads to a dramatic decrease in yield, but at least the increase in quality renders the material usable within contemporary genomics workflows that include library construction and cDNA sequencing.

## Conclusion

While there is undoubtedly scope for the further improvement of RNA yields from lichen using inexpensive methods, we have shown a simple approach for the extraction of RNA of sufficiently high quality for reverse transcription and cDNA library construction. The total RNA extracted in this fashion has successfully been used for cDNA library construction and the quality of the resulting sequences has been validated and has yielded the first reindeer lichen ESTs. The commercial extraction kit and our preferred CTAB - clean-up method are both equally demanding in terms of hands-on laboratory time, and future improvements could be made to reduce this.

A preliminary analysis of these first ESTs reveals that there is an expected bias towards sequences of a fungal origin, although sequences more related to algal or plant species are also observed (Additional file 1). The many apparently novel sequences suggests that the transcriptome sampling of lichens may be used to identify novel genes and may be used to characterise the molecular mechanisms that allow lichens to survive some of the harshest environments.

## Competing interests

The authors declare that they have no competing interests.

## Authors' contributions

SJ conducted the experiments and drafted the manuscript. KJL conducted cDNA library construction. SR performed the EST sequence analysis and drafted the manuscript.

## Appendix

### Abbreviated protocol for the two step CTAB - RNA cleanup protocol for RNA extraction from Cladonia rangiferina

1. Grind 1 g of lichen tissue in liquid nitrogen with mortar and pestle

2. Add 10 ml of prewarmed (65°C) extraction buffer (2% CTAB, 2% polyvinylpyrrolidone MW 40 000, 200 mM Tri-HCl pH 8.0, 25 mM EDTA, 2 M NaCl, 0.5 g/l spermidine) with 2% β-mercaptoethanol

3. Extract twice with phenol-chloroform (1:1)

4. Add equal volume of NTES buffer (1 M NaCl, 0.5% SDS, 10 mM, Tris-HCl pH 8.0, 1 mM EDTA) and chloroform (1:1) to the supernatant

5. Precipitate RNA with ethanol and dissolve in water

Clean RNA using QIAgen RNeasy columns according to manufacturer's instructions

## Supplementary Material

Additional file 1 ***Cladonia rangiferina *unigene sequences with a BLASTX match**. The collated results from the *Cladonia rangiferina *unigene sequences having BLASTX matches against protein sequences in the NCBI NonRedundant protein sequence database exceeding the arbitrary expectation value of 1e-10. For each sequence is shown the NCBI accession and species of origin for the best matching sequence and the number of distinct plant, and fungal species (satisfying the 1e-10 expectation value threshold) matched. This can be used to identify tentative fungal and plant-specific sequences. The sequences flagged with a '*' appear fungal specific, while sequences flagged with the '†' have plant or algal sequence matches and no fungal matches, but may also match sequences of animal, protist or bacterial origin. Click here for file
